# Water flow buffers shifts in bacterial community structure in heat-stressed *Acropora muricata*

**DOI:** 10.1038/srep43600

**Published:** 2017-02-27

**Authors:** Sonny T. M. Lee, Simon K. Davy, Sen-Lin Tang, Paul S. Kench

**Affiliations:** 1 School of Environment, The University of Auckland, Private Bag 92019, Auckland, New Zealand; 2 Department of Medicine, The University of Chicago, Chicago, IL, United States; 3 School of Biological Sciences, Victoria University of Wellington, Kelburn Parade, Wellington, New Zealand; 4 Microbial Lab, Biodiversity Research Center, Academia Sinica, Taipei, 115, Taiwan

## Abstract

Deterioration of coral health and associated change in the coral holobiont’s bacterial community are often a result of different environmental stressors acting synergistically. There is evidence that water flow is important for a coral’s resistance to elevated seawater temperature, but there is no information on how water flow affects the coral-associated bacterial community under these conditions. In a laboratory cross-design experiment, *Acropora muricata* nubbins were subjected to interactive effects of seawater temperature (27 °C to 31 °C) and water flow (0.20 m s^−1^ and 0.03 m s^−1^). In an *in situ* experiment, water flow manipulation was conducted with three colonies of *A. muricata* during the winter and summer, by partially enclosing each colony in a clear plastic mesh box. 16S rRNA amplicon pyrosequencing showed an increase in the relative abundance of *Flavobacteriales* and *Rhodobacterales* in the laboratory experiment, and *Vibrio* spp. in the *in situ* experiment when corals were exposed to elevated temperature and slow water flow. In contrast, corals that were exposed to faster water flow under laboratory and *in situ* conditions had a stable bacterial community. These findings indicate that water flow plays an important role in the maintenance of specific coral-bacteria associations during times of elevated thermal stress.

The coral microbiome is a collective community of microorganisms that are associated with the coral host^1^,^2^. These coral-specific microbial communities are hypothesized to have important physiological and ecological roles on coral reefs, such as fixing nitrogen and carbon^3^, removing nitrogenous waste from the coral host^4^, and protecting the coral host against opportunistic pathogens^5^. Recent studies have provided compelling evidence that coral-microbial associations are more complex than previously believed, and are significantly influenced by factors specific to the physiological^6^,^7^ and physical environment of the coral host^1^,^8^,^9^. Physical factors such as temperature, light and water flow vary significantly over stony coral reefs, and have an impact on both the host and its microbial communities^10^,^11^,^12^,^13^. Therefore, understanding the interaction between the environment, host coral and its associated microbes is essential for understanding the health and function of coral reefs.

Within the coral holobiont, composition of the microbial community is highly influenced by microhabitat, such as the surface mucus layer (SML) and coral tissues^14^. The SML is an important interface between the coral and the surrounding seawater^15^,^16^,^17^,^18^, and the mucus is a carbon-rich substance that serves as an important substrate for bacterial growth^17^,^19^. There are various properties of the coral mucus layer that protect the coral host from pathogenic bacteria, such as: acting as a physical barrier^20^; sloughing of potential pathogens^21^,^22^,^23^; and serving as a medium for anti-bacterial allelochemicals^5^,^24^,^25^. In addition, it has been hypothesized that the coral host may be able to control the bacterial communities that inhabit the SML in order to cultivate the growth of beneficial bacteria, such as nitrogen fixers or those that inhibit potential pathogens^26^. Similarly, the SML may protect the coral host from invasive microbes simply as a result of the native microbiota occupying certain niches within this layer^27^,^28^. On the other hand, bacterial communities within the coral tissues are significantly different from those in the SML^29^,^30^,^31^,^32^. Studies have shown that opportunistic and pathogenic bacteria are present within healthy coral tissues and the SML^27^,^33^, and that microorganisms will take advantage of a stressed coral host and proliferate in abundance^13^,^34^. Due to the distinct differences between the SML and coral tissues, it is important to analyze the coral-associated microbiota within the different layers of coral colonies separately, in order to understand the role of the microbial communities in coral diseases.

Environmental stressors that impact host physiology also change the microbial environment and the microbiome. Bourne *et al*.^35^ showed that microbial communities in bleached corals were different from those in healthy corals in the months before bleaching and following recovery. Similarly, laboratory experiments have demonstrated that there is a change in the coral’s microbiota before there are any visual signs of stress^13^. Microbial metabolism and physiology are directly affected by the same environmental disturbances that alter the host’s metabolism^36^. Environmental stressors will not only change the coral host’s physiological health and microbial structure, but also the interaction between the coral and the microbial community within each microhabitat. Various studies have demonstrated that health of the coral host deteriorates when seawater temperature increases, and results in greater opportunities for infection by coral-associated pathogens^7^,^13^,^35^,^37^,^38^,^39^,^40^.

Water is constantly moving at various temporal and spatial scales in the reef environment, and has a strong effect on the coral host and microbial communities^41^. Several studies have shown a positive correlation between water flow and parameters such as photosynthetic rate^42^, coral growth^43^, phosphate uptake^44^, amino acid accumulation under ultraviolet exposure^45^,^46^, mitigation of oxidative stress^47^, and survival under high irradiance and sea-surface temperatures^48^. Nakamura and Yamasaki^49^ found that with higher water flow, bleaching was suppressed under high seawater temperatures in *Pocillopora damicornis* and *Stylophora pistillata*. While there has been a tremendous amount of research on the effects of water flow on coral bleaching^50^, there have been no attempts to date to analyze bacterial community shifts between the SML and tissues of the coral host under the interactive effects of water flow and increasing thermal stress. Bacteria live in dynamic fluid environments^51^,^52^, and water flow can have an effect on microbial concentrations^11^ and processes, such as nutrient uptake^51^,^52^,^53^ and infection^54^. Furthermore, the hydrodynamic environment will directly affect bacterial fitness^55^ and foraging techniques^53^.

Here we characterized the combined effects of water flow and thermal stress on the coral (*Acropora muricata*) holobiont, and simultaneously measured the shifts in bacterial diversity in the coral SML and tissues under both laboratory and *in situ* conditions. Profiles of the coral-associated bacterial communities were analyzed, with a further emphasis on investigating the relative changes in *Vibrio* spp. as potential pathogenic bacteria, to illustrate the possibility of a shift to a pathogen-dominated community when the coral host is under stress. We demonstrate that elevated water flow can buffer the effects on bacterial community shifts within the mucus and tissue layers.

## Results

### Effects of elevated temperature on coral health

#### Laboratory experiment

**Figure 1:**
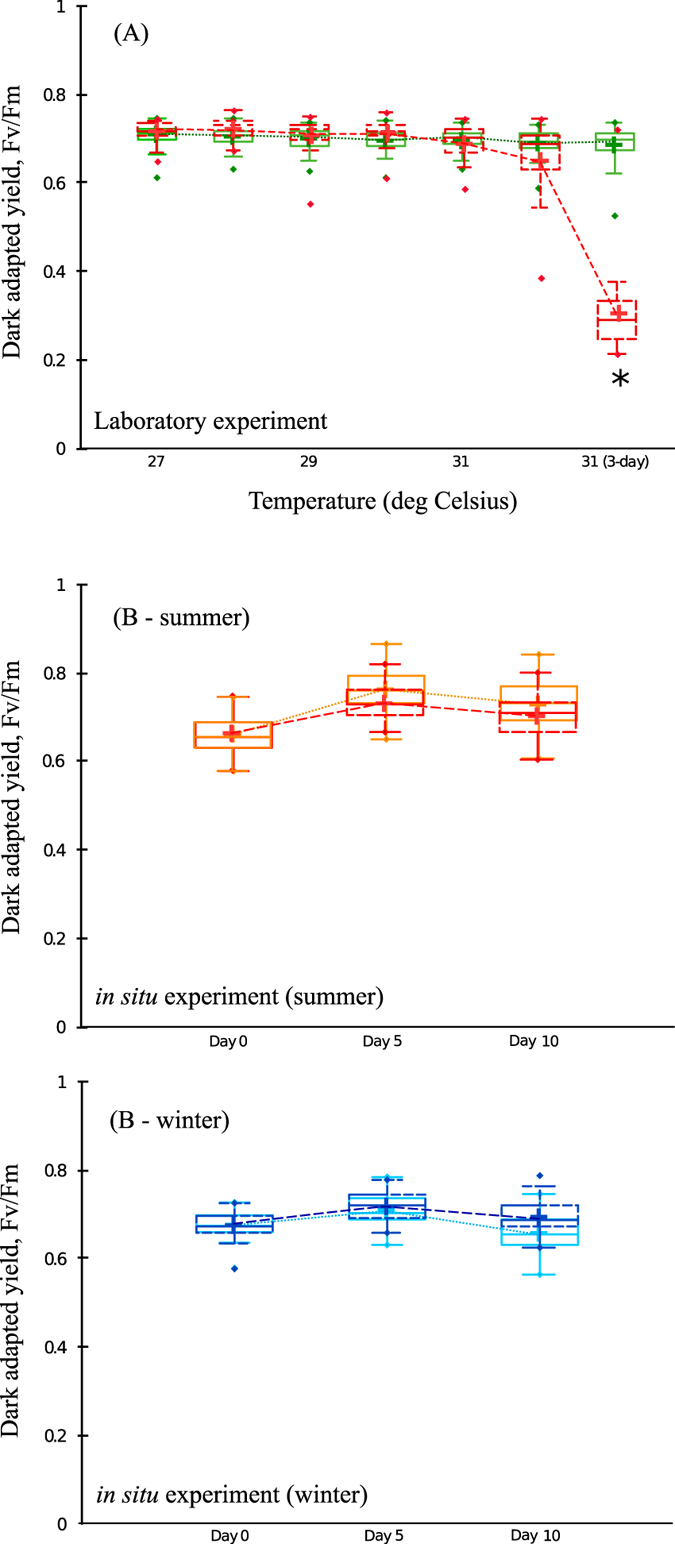
Photochemical efficiency (Mean ± S.D.), measured with a Diving PAM fluorometer, for laboratory (**A**) and *in situ* field (**B**) experiments. Laboratory – Green box represents groups subjected to high water flow and the low water flow with control temperature treatment, and red box represents groups subjected to low water flow and high temperature. The temperature in the treatment tanks was increased 1 °C *per* day from 27 °C to 31 °C, after which it was kept constant at 31 °C. High water flow was ~0.20 m s^−1^ and low water flow was ~0.03 m s^−1^. *P < 0.05. *In situ* – (Summer/Winter) light blue/light red box represents un-enclosed, and dark red/dark blue box represents enclosed treatments. Figures show mean with 75% quantile, and S.D. (whiskers), and outliers (•).

No visual signs of stress or bleaching were evident for coral nubbins that were exposed to high water flow irrespective of temperature, or low water flow at the control temperature. However, coral nubbins that were subjected to low water flow × high temperature showed a strong decline in the photochemical efficiency (F_v_/F_m_) of their symbiotic dinoflagellates after exposure to 31 °C for three days (F_v_/F_m_ = 0.294 ± 0.095, Mean ± S.D., PERMANOVA, Pseudo-F_1,8_ = 7.843, *P* = 0.005, Fig. 1A).

#### *In situ* experiment

There was no significant difference in F_v_/F_m_ of the dinoflagellate symbionts of all three *Acropora muricata* colonies (enclosed and un-enclosed), regardless of season (summer, winter) (PERMANOVA, Pseudo-F_1,8_ = 0.843, *P* = 0.62; Fig. 1B). There was a significant difference between the water temperature in the summer (28.86 ± 1.06 °C) and winter (23.21 ± 1.36 °C) (one-way ANOVA, F_1,18_ = 239.3, P < 0.001, Supplementary Figure S1A). There was no difference in irradiance between the parts of the coral that were enclosed and un-enclosed (one-way ANOVA, F_1,38_ = 0.467, P = 0.498, Supplementary Figure S1B). Although the irradiance on Day 1 and 2 of the experiment in winter was similar to that experienced in summer, Day 3–10 of the winter experiment (with cloudy skies and rainy days) experienced considerably lower irradiances (Supplementary Figure S1B). The portion of the corals that was enclosed experienced reduced water flow (one-way ANOVA, F_1,38_ = 5.867, P < 0.020, Supplementary Figure S1C). During the summer, the portion of corals enclosed experienced water flow of 15.50 ± 5.74 cm s^−1^, while the unenclosed portion experienced water flow of 30.19 ± 6.06 cm s^−1^. During the winter, the portion of coral enclosed experienced lower water flow than the portion that was unenclosed (enclosed: 16.18 ± 8.11 cm s^−1^, unenclosed: 33.87 ± 7.16 cm s^−1^; Supplementary Figure S1C).

### Bacterial community composition

#### Laboratory experiment

**Figure 2:**
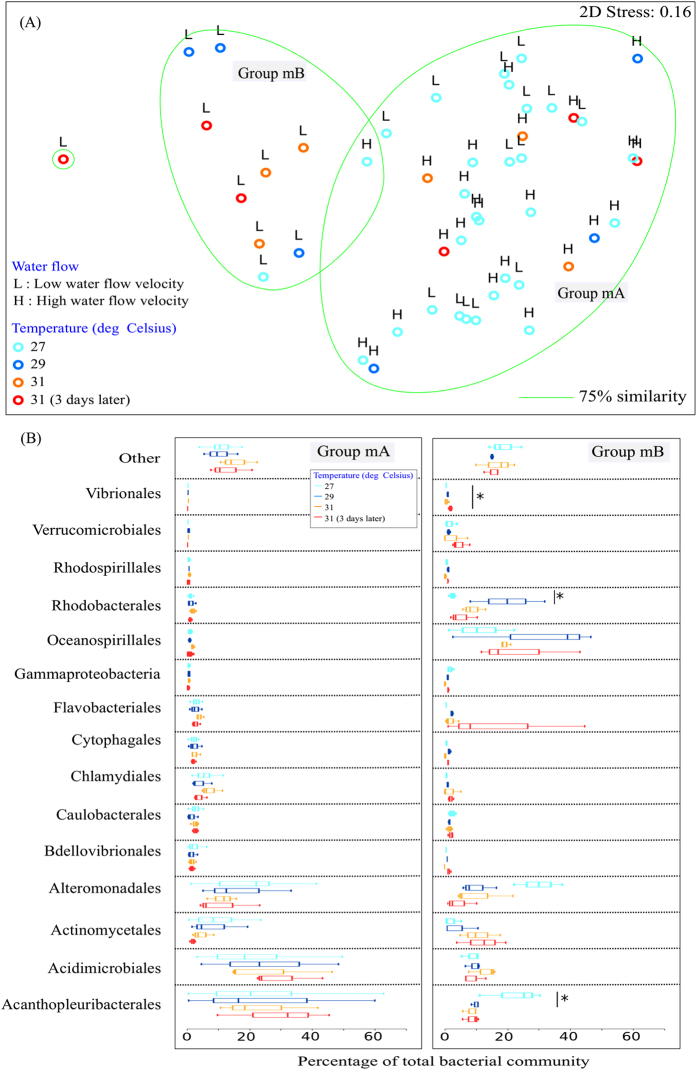
Bacterial community composition of coral mucus in the laboratory experiment. (**A**) nMDS cluster analysis of coral mucus bacterial community profiles showed two distinct groups – mA and mB. (**B**) Bacterial community composition (order level) of Group mA and mB. OTUs with a mean relative abundance of less than 1% are combined in the ‘Others’ category. Figures show mean with 75% quantile, and S.D. (whiskers). *P < 0.05.

**Figure 3:**
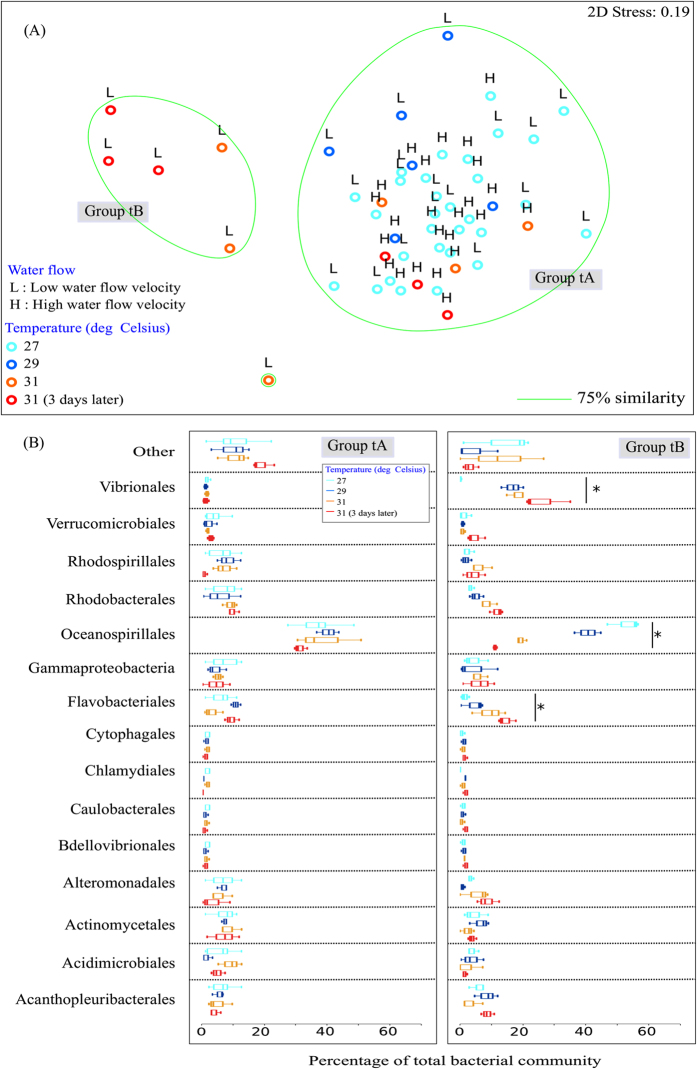
Bacterial community composition of coral tissue in the laboratory experiment. (**A**) nMDS cluster analysis of coral tissue bacterial community profiles showed two distinct groups – tA and tB. (**B**) Bacterial community composition (order level) of Group tA and tB. OTUs with a mean relative abundance of less than 1% are combined in the ‘Others’ category. Figures show mean with 75% quantile, and S.D(whiskers). *P < 0.05.

Non-metric multidimensional scaling (nMDS; 2D Stress: 0.16; 0.19 with Bray-Curtis similarity index) on square-root normalized values of the bacterial community profiles in the mucus and tissues separated the samples into two distinct groups – Group mA and mB (Fig. 2A), and Group tA and tB (Fig. 3A). Groups mA and tA consisted of SML and tissue samples in the f_High_t_High_, f_High_t_Control_ and f_Low_t_Control_ treatments, while samples in the f_Low_t_High_ treatment were distinctly different in the mB and tB groups (Figs 2A and 3A). There was a significant difference in bacterial community composition in the surface mucus layer (SML) (PERMANOVA, Pseudo-F_3,40_ = 3.082, *P* = 0.001; Fig. 2A) and tissue layer (PERMANOVA, Pseudo-F_3,40_ = 3.637, *P* = 0.001; Fig. 3A) between the two groups (Group mA *vs.* mB, and Group tA *vs.* tB). Acanthopleuribacterales was responsible for the largest fraction (SIMPER, 22.10%) of the SML bacterial composition differences between the two groups, while Oceanospirillales contributed to 19.86% (SIMPER) of the differences in the tissue layer bacterial composition.

Sequences affiliated with Acanthopleuribacterales were high in relative abundance in the mB group (22.2 ± 10.0%, Mean ± S.D.) at 27 °C, but decreased significantly to 8.0 ± 2.6% after exposure to 31 °C for an extended period of time (t(4) = 4.222, *P *=* *0.002). Members of the Rhodobacterales increased significantly in relative abundance (t(4) = −2.852, *P* = 0.046) from 27 °C (2.2 ± 0.9%) to 29 °C (19.6 ± 12.1%), but then decreased rapidly in relative abundance at 31 °C (9.2 ± 3.7%), and continued to decrease in relative abundance to 4.6 ± 4.6% when this high temperature persisted (Fig. 2B). Members of the order Vibrionales increased their relative abundance significantly (t(4) = −3.423, *P* = 0.007) from 0.1 ± 0.1% at 27 °C to 0.8 ± 0.6% at 31 °C, and further increased their relative abundance to 1.0 ± 0.5% after being exposed to 31 °C for an extended period of time (Fig. 2B).

The bacterial community in the coral tissues in Group tA was dominated by members of the Oceanospirillales (Fig. 3B). The relative abundance of Oceanospirillales remained consistent in the tA group (27 °C – 36.9 ± 7.4%; 29 °C – 39.7 ± 3.5%; 31 °C – 38.3 ± 10.5%; 31 °C, 3 days later – 31.6 ± 2.1%), regardless of temperature change. However, in the tB group, the relative abundance of Oceanospirillales decreased (t(4) = 13.667, *P* = 0.000) from 52.7 ± 5.3% at 27 °C to 10.6 ± 0.6% after the ended period at 31 °C (Fig. 3B). In the tB group, sequences affiliated with Vibrionales were low in relative abundance at 27 °C (0.2 ± 0.2%), but started to increase as the temperature increased (29 °C – 16.7 ± 3.5%, 31 °C – 18.3 ± 2.8%, 31 °C, 3 days later – 26.0 ± 7.8%; t(4) = −5.727, *P* = 0.005). Members of the Flavobacteriales also started to become more prominent in the tB group at high temperature (31 °C, 3 days later– Flavobacteriales: 14.0 ± 2.9%, t(4) = −6.720, *P* = 0.003; Fig. 3B).

#### *In situ* experiment

**Figure 4:**
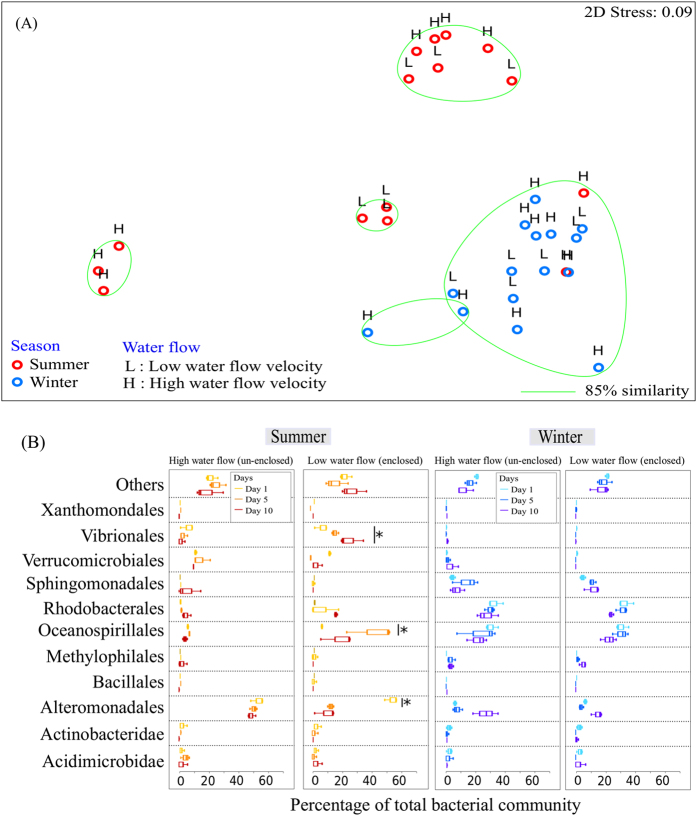
Bacterial community composition of coral mucus in the *in situ* field experiment. (**A**) nMDS cluster analysis of coral mucus bacterial community profiles did not show any distinct pattern. (**B**) Bacterial community composition (order level) of samples in high water flow (un-enclosed) and low water flow (enclosed) on Days 1, 5 and 10 during the summer and winter. OTUs with a mean relative abundance of less than 1% are combined in the ‘Others’ category. Figures show mean with 75% quantile, and S.D(whiskers). *P < 0.05.

**Figure 5:**
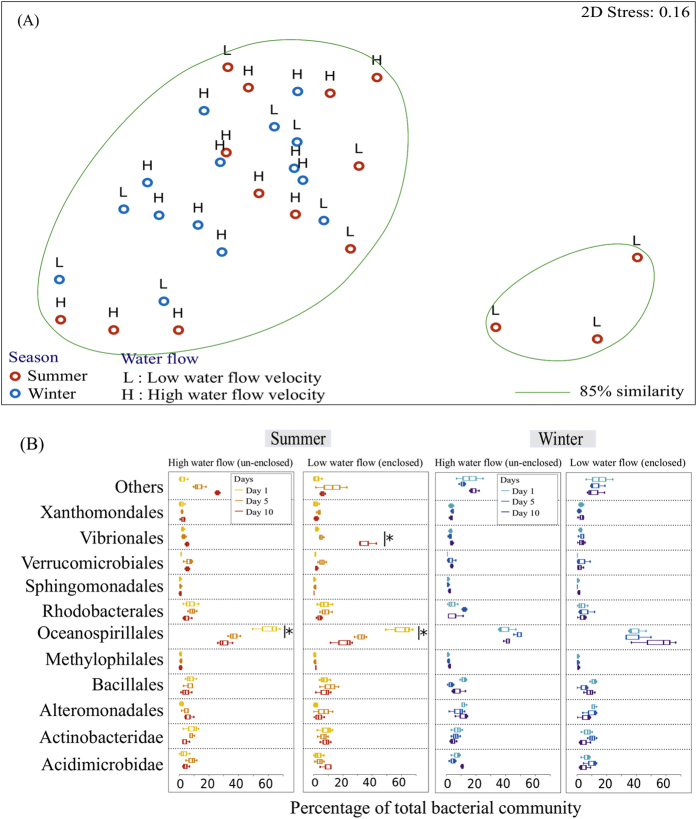
Bacterial community composition of coral tissues in the *in situ* field experiment. nMDS cluster analysis of coral tissue bacterial community profiles did not show any distinct pattern. (**B**) Bacterial community composition (order level) of samples in high water flow (un-enclosed) and low water flow (enclosed) on Days 1, 5 and 10 during the summer and winter. OTUs with a mean relative abundance of less than 1% are combined together in the ‘Others’ category. Figures show mean with 75% quantile, and S.D. (whiskers). *P < 0.05.

There was a significant effect of season (winter *vs.* summer) (PERMANOVA, Pseudo-F_1,29_ = 7.024, *P* = 0.002; Fig. 4A) but not water flow (enclosed *vs*. un-enclosed) (PERMANOVA, Pseudo-F_1,29_ = 0.979, *P* = 0.385; Fig. 4A) on the SML bacterial community composition of the coral colonies. Oceanospirillales contributed the largest percentage to the differences in the bacterial composition between seasons (SIMPER, 45.71%), and between different flow treatments (SIMPER, 20.98%). In the coral tissues, there was a weak significant difference in the bacterial communities between the enclosed and un-enclosed portions of the colony in different seasons (PERMANOVA, Pseudo-F_1,29_ = 2.178, *P* = 0.042; Fig. 5A). However, there was no significant difference in the tissue layer bacterial community composition between the enclosed or un-enclosed portions of the three colonies (PERMANOVA, Pseudo-F_1,29_ = 0.486, *P* = 0.879; Fig. 5A), nor was there any significant difference in the bacterial community between winter and summer (PERMANOVA, Pseudo-F_1,29_ = 1.620, *P* = 0.118; Fig. 5A). As in the SML, Oceanospirillales contributed the largest differences between the treatments (SIMPER, between seasons – 25.35%; between water flow treatments – 28.16%).

Oceanospirillales and Alteromonadales were the prominent taxa in the *A. muricata* mucus community during both winter and summer (Fig. 4B). In the summer, members of the Oceanospirillales were low in relative abundance at the start of the experiment (5.5 ± 0.3%) and remained consistent in the unenclosed treatment, but increased significantly in the enclosed treatment (Day 5: 42.8 ± 16.5%; Day10: 35.3 ± 17.8%; t(4) = −2.899, *P* = 0.044; Fig. 4B). During summer, members of the Alteromonadales were prominent in the coral SML at the start of the experiment (50.1 ± 10.2%), but decreased in relative abundance in the enclosed treatment (Day 5: 12.9 ± 2.0%; Day 10: 9.6 ± 7.2%; t(4) = 8.963, *P* = 0.001; Fig. 4B), while the relative abundance remained high in the un-enclosed treatment. Members of the Vibrionales were not retrievable from the coral mucus during winter, but started to become more prominent by Day 10 during the summer survey. In the summer, at the end of the experiment (Day 10), the relative abundance of Vibrionales in the SML was higher in the enclosed portion (21.5 ± 9.2%; t(4) = 2.924, *P* = 0.015) than the un-enclosed portion (1.5 ± 2.4%) of the coral colony (Fig. 4B).

Members of the Oceanospirillales were the most prominent taxa in the coral tissue community during both summer and winter (Fig. 5B). The relative abundance of Oceanospirillales was 35.1 ± 16.4% in summer and 44.2 ± 9.6% in winter (Fig. 5B), and there was a significant decrease in relative abundance of Oceanospirillales in summer (unenclosed: t(4) = 4.74, *P* = 0.009; enclosed: t(4) = 5.622, *P* = 0.005) but not in winter. Members of the Vibrionales were low in relative abundance throughout winter (2.6 ± 1.5%) and at the start of summer (2.6 ± 0.9%; Fig. 5B). However, by Day 10 in summer, the relative abundance of Vibrionales in the coral tissues was higher in the enclosed portion (35.7 ± 6.5%; t(4) = 8.797, *P* = 0.001) than the un-enclosed portion (6.7 ± 4.5%) of the coral colonies (Fig. 5).

#### Vibrio component of the bacterial community

**Figure 6:**
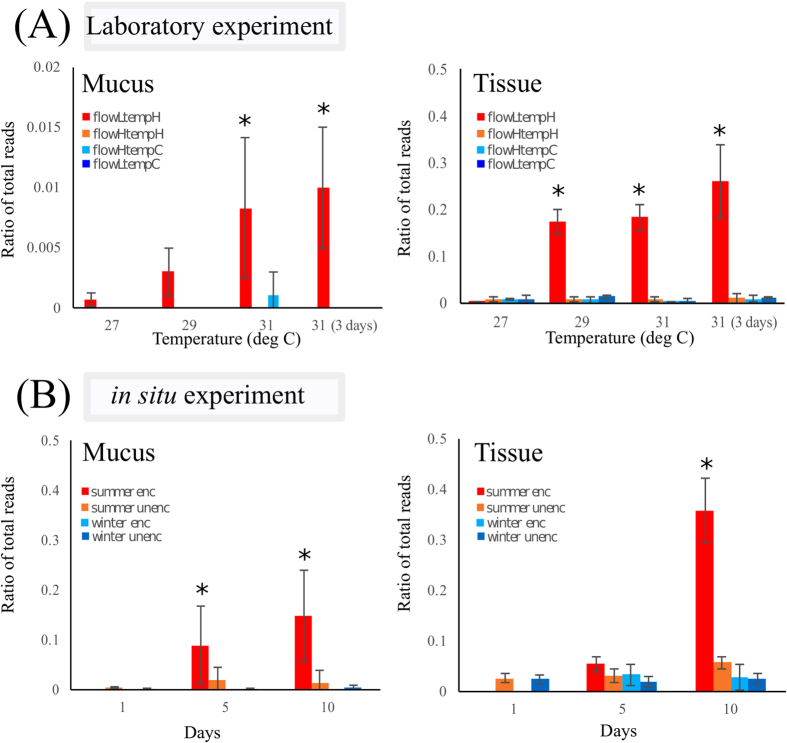
Bacterial community shift (Mean ± S.D.) of *Vibrio* spp. in (**A**) coral mucus and tissues when the temperature was increased in the laboratory from 27 °C to 31 °C, and held at this latter temperature for an extended period. The four treatments for temperature and water flow are high water flow × high temperature (flowHtempH), high water flow × control temperature (flowHtempC), low water flow × high temperature (flowLtempH) and low water flow × control temperature. (flowLtempC). (**B**) Relative abundance of *Vibrio* spp. in coral mucus and tissues during summer and winter, enclosed (enc) and un-enclosed (unenc) treatments in the field. Values shown are the relative abundance of *Vibrio* spp. as a ratio of the total community sequences. *P < 0.05.

Further analysis focused on *Vibrio* spp., to explore the changes in relative abundance of these potential pathogens when the coral host is under thermal stress. In the laboratory experiment, there was a significant change in the *Vibrio* spp. relative abundance in both the SML and tissues when the coral host was subjected to different water flow and temperature regimes (mucus: PERMANOVA, Pseudo-F_3,47_ = 7.556, *P* = 0.001; tissue: PERMANOVA, Pseudo-F_3,47_ = 23.473, *P* = 0.001; Fig. 6A). Pairwise analysis revealed that the most significant changes in relative abundance of *Vibrio* spp. were when the coral samples were exposed to low water flow (mucus: between 27 °C and 31 °C-3 days later, t_16_ = 7.580, *P* = 0.004; tissue: between 27 °C and 31 °C, t_16_ = 7.531, *P* = 0.001; Fig. 6A). In the field experiment, there was a small but significant change in the *Vibrio* spp. relative abundance associated with the coral mucus (PERMANOVA, Pseudo-F_1,29_ = 7.266, *P* = 0.014; Fig. 6B), but there was no significant change in the relative abundance of *Vibrio* spp. in the tissues (PERMANOVA, Pseudo-F_1,29_ = 2.240, *P* = 0.153; Fig. 6B) when the corals were subjected to the interactive effects of water flow and season. However, sequences affiliated with *Vibrio* spp. were significantly different between the summer and winter (mucus: PERMANOVA, Pseudo-F_1,29_ = 25.916, *P* = 0.001; tissue: PERMANOVA, Pseudo-F_1,29_ = 9.521, *P* = 0.007; Fig. 6B).

## Discussion

Our data provide evidence that increased water flow influences the bacterial community within the coral surface mucus (SML) and tissue layers. In particular, the laboratory experiment provided evidence that increased water flow can limit the capacity of potential pathogens to proliferate, while *in situ* observations confirmed that water flow can influence microbial community composition and, potentially, coral health.

Our results demonstrate the cascading effect of water flow and rising water temperature on coral health, and subsequently on the bacterial community in the SML and coral tissues. In this study, the deterioration in coral physiology was only prominent in the f_Low_t_High_ treatment (Group mB and tB; Figs 2 and 3), both visually and photochemically. Coral samples in Group mA, which were exposed to the f_High_t_High_, f_High_t_Control_ and f_Low_t_Control_ treatments (Group mA and tA; Figs 2 and 3), had a relatively stable bacterial community in the SML that was dominated by members of the Acanthopleuribacterales, Acidimicrobiales and Actinomycetales, with low relative abundances of Oceanospirillales and Flavobacteriales. In contrast, members of the Oceanospirillales made up a high percentage of the bacterial community in coral tissues under the f_High_t_High_, f_High_t_Control_ and f_Low_t_Control_ treatments, which is in agreement with previous studies that found Oceanospirillales to be dominant in the tissues of healthy corals^1^,^56^,^57^,^58^,^59^. Regardless of whether the water temperature was kept at a constant 27 °C or increased to a stressful 31 °C, the stable bacterial community within the SML and tissues suggests that turbulence created by high water flow may help maintain the coral host’s physiological health^60^. Interestingly, with slow water flow, we observed a decrease in the relative abundance of Rhodobacterales in the SML as the seawater temperature increased, with a consecutive increase in the relative abundance of Rhodobacterales in the tissue layer as the coral was exposed to high temperature for an extended period of time. We speculate that, as the seawater temperature increases, the coral SML undergoes changes in mucus composition^61^,^62^, mucus structure^21^ and the amount of mucus produced^63^,^64^, allowing members of the Rhodobacterales to pass through the mucus barrier and invade the tissues; visual cellular assessments, such as *via* fluorescence *in situ* hybridization (FISH) or transmission electron microscopy (TEM), will help to elucidate this matter. Wooldridge^61^ has pointed to the possibility of a change in coral mucus composition during bleaching, while both Fitt *et al*.^63^ and Piggot *et al*.^64^ demonstrated that internal mucus reservoirs in the coral tissues are depleted during bleaching, resulting in very low levels of mucus release. Therefore, a loss in the functional quality of the coral mucus could enable Rhodobacterales to overcome the coral host defenses provided by the mucus, and to invade the underlying tissues. Furthermore, several studies have showed that the coral SML not only serves as a medium in which anti-bacterial allelochemicals may be exuded^5^,^24^,^65^,^66^,^67^, but it also houses a native microbiota that can regulate the population of pathogens within the SML through extracellular activities^68^. Rhodobacterales have previously been reported to be present in corals infected by white plague disease^69^. It is plausible that potential pathogens in the class Rhodobacterales had different patterns of enzymatic regulation and activities for the sugars present in the SML^70^, and were able to outcompete the native microbiota^71^,^72^ within the coral mucus and tissues at higher temperatures. Alternatively, the poor health of corals arising from thermal and hence oxidative stress^47^ could provide an opportunity for pre-existing members of the microbial population to proliferate and, take advantage of their weakened host. Further investigation of the prominent bacterial species in the class Rhodobacterales and their metabolic pathways will provide insights into the proliferation of the bacteria in the SML and tissues at high temperatures.

Water flow can reduce the level of bleaching under natural conditions^73^. By bridging the laboratory and field experiments, results from this study could provide valuable information on the impacts of water flow on coral-associated bacterial communities in the SML and tissues. Results from the *in situ* experiment did not exhibit any clear trends (Figs 4 and 5), however, suggesting that there were complex interactions between more environmental factors than just water flow and temperature^33^,^38^,^74^,^75^. Various studies have reported that the diversity of coral-associated bacteria is affected by geographical locations^57^,^74^,^76^,^77^,^78^,^79^ and seasons^74^,^80^,^81^. For example, in Taiwan, Hong *et al*.^74^ provided a comprehensive analysis of the microbial communities associated with *Stylophora pistillata* and found that, not only are the bacterial profiles highly variable among individual coral colonies, but the bacterial community population also changes rapidly with geographical location and season. In this study, our results suggest that the range of water temperatures at the test-site in Kenting during the experimental periods (23–28 °C) was probably not significant enough to trigger any macroscopic impact, such as visual bleaching, on the coral colonies. Perhaps larger temperature changes might elicit a more direct effect on the colonies. Another important environmental factor that could affect the *in situ* experiment is the presence of the Kuroshio Current. The Kuroshio Current is a high speed tropical current from the Philippines, flowing past subtropical Taiwan and Okinawa to the temperate region of Japan^82^,^83^. The Kuroshio Current may have supplied sufficient water movement around the coral colonies, thus limiting the effects of reduced water flow around the enclosed portions. In this study, portions of the coral colonies that were enclosed experienced water-flow velocities of 16.18 ± 8.11 cm s^−1^ in winter and 15.50 ± 5.74 cm s^−1^ in summer. In comparison, the water flow velocities in the *in situ* experiment were much higher than that in the laboratory experiment (~3 cm s^−1^), and the reduced water flow rate in the *in situ* study was probably still high enough to provide beneficial effects against elevated temperature. Another potential contributing factor that may have an impact on the results from the laboratory and field experiments was our method of mucus collection. In particular although the method used in both the aquaria and *in situ* studies was consistent, the discarding of mucus for an initial 30 s might result in loss of certain microbial communities, since corals use sloughing as a mechanism to remove potential pathogens^21^,^22^,^23^. Nevertheless, our results provide evidence that, irrespective of temperature, reduced water flow around the enclosed portions of the coral colonies may allow potential pathogens to become more dominant.

Sufficient water flow around coral colonies will buffer the shift of native microbiota in the coral SML and tissues to a pathogen-dominated community. In our laboratory experiment, stress of *A. muricata* was only visible in the f_Low_t_High_ treatment when the coral samples were under high temperature for an extended period of time, while corals in the other treatments showed no signs of bleaching. The associated increase in the relative abundance of *Vibrio* spp. in the SML and tissues at high temperatures/low flow suggested either a proliferation or migration of these pathogens within the two coral compartments (i.e. SML and tissues), with proliferation seeming more likely given the deterioration in health of the coral (Fig. 6). In the field experiment, the appearance of *Vibrio* spp. in the coral SML and tissues during summer may provide some insight into of importance of water flow as a factor in determining a coral’s resistance to disease. In the field experiment, there was no significant difference in photosynthetic health between the portions of corals that experienced reduced water flow *versus* those that experienced high water flow. However, at the end of the experiment in summer, sequences affiliated with *Vibrio* spp. were higher in relative abundance in the enclosed coral mucus and tissue samples than the open samples. These results suggest that the bacterial community composition in the SML and tissues was starting to shift, and that *Vibrio* spp. that are potentially pathogenic were starting to be more prominent in the enclosed portions of the coral colonies when the seawater temperature increased. This is not surprising given that coral-associated microbial community changes have been observed even before the coral host shows any visual signs of stress^13^,^35^. Various studies have shown that members of the genus *Vibrio* – *Vibrio shiloi*^84^ and *V. coralliilyticus*^13^,^37^,^40^,^85^ - proliferate in stressed and diseased corals at seawater temperatures above 27 °C. Given that all other environmental factors were similar, a 10-fold difference in relative abundance of *Vibrio* spp. between the enclosed and open portions of the coral colonies suggests that the slower water flow might cause the corals to experience more stress from the elevated seawater temperature during summer. Glasl *et al*.^23^ found an increase in prevalence of opportunistic and potentially pathogenic bacteria (Verrucomicrobiaceae and Vibrionaceae) in aged mucus of *Porites astreoides*. Adequate water flow around corals is a critical factor in maintaining a stable mucus community^21^,^22^,^23^, and thus findings from this and Glasl *et al*.^23^ provide a possible mechanistic understanding of the important role of water flow in determining coral health. Results from both the laboratory and field experiments suggest that increased seawater temperature is the main driver for the changes in coral host health, while water flow is a secondary factor that may increase a coral’s resistance in times of thermal stress. Our field experiment results were much less conclusive than those from the laboratory, partly because the water flow differences *in situ* were not as great and the temperature never reached that of the laboratory scenarios (31 °C). Moreover, well-controlled aquarium experiments are inherently different from field studies, where there are often unforeseen stresses and consequences for the coral microbiome^86^.

Our findings demonstrate that increased water flow not only serves as an ameliorator for thermally-stressed corals, but also prevents potentially harmful shifts in the coral’s microbial community. In particular, both our controlled laboratory and field experiments showed that sufficient water flow around coral colonies can prevent a shift in the bacterial community composition of the SML and potentially prevent the bacterial community in the coral’s tissues from changing to a pathogen-dominated state. More importantly, thermally induced changes to the bacterial community under low flow involved a dramatic increase in the relative abundance of pathogens, such as *Flavobacteriales, Rhodobacterales* and *Vibrio* spp. While further long-term field studies with more replicates are necessary to explore other factors that may impact coral health and the coral-associated bacterial community, our results highlight the need to consider water flow when assessing the health of reef corals under thermal stress, and in particular shifts in the community composition of the coral holobiont.

## Methods

This study was conducted at the National Museum of Marine Biology and Aquarium (NMMBA), Taiwan, and in Kenting, Taiwan (21°57′N, 120°46′E), during the winter and summer sampling seasons of December 2013 and June 2014.

### Laboratory experiment

To examine the effects of water flow on the bacterial community composition in heat-stressed corals, a set of laboratory-based studies was performed. Coral nubbins, approximately 2 cm in length, were collected from Kenting National Park, Nan-wan, Taiwan (21°57′N, 120°44′E) on June 20, 2014. A total of 100 coral nubbins were collected from five different *Acropora muricata* colonies at 8–10 m depth. The collected samples were acclimated in a 0.2 μm filtered seawater flow-through tank for 30 days, with a constant water temperature of 27 °C and an irradiance of ~150 μmol photons m^−2^ s^−1^ on a 12 h light/dark cycle (HQI metal halide lamp). After the acclimation, 96 coral nubbins were distributed randomly into 12 different treatment tanks, all of which contained 0.2 μm filtered seawater, and were irradiated at ~150 μmol photons m^−2^ s^−1^ by a HQI metal halide lamp. There were three replicates of four different treatments – high water flow × high temperature (f_High_t_High_), high water flow × control temperature (f_High_t_Control_), low water flow × high temperature (f_Low_t_High_) and low water flow × control temperature. (f_Low_t_Control_). Tanks which had high water flow had a pump attached, moving the water at a speed of ~0.20 m s^−1^, while tanks that had low water flow had a diffuser which slowed down the water flow to ~0.03 m s^−1^. Water flow velocities (~0.20 m s^−1^ and ~0.03 m s^−1^) used in this study was based on previous studies done by Nakamura and van Woesik^48^ showing requirements of water flow rates for *Acropora digitifera* survivorship under high temperature, and *in situ* field measurements at the locations where the samples were collected (Supplementary Figure S1C). Control temperature tanks were kept at a constant temperature of 27 °C (±1 °C), while water temperature in the other six treatment tanks was raised 1 °C *per* day from 27 °C to 31 °C (±1 °C), after which it was kept constant.

One coral nubbin and one liter of seawater were collected from each tank on the days when the respective treatment tank temperatures were 27 °C, 29 °C, 31 °C and 31 °C (3 days later). The laboratory experiment was concluded on the seventh day because coral samples showed signs of tissue degradation after maintaining the temperature at 31 °C for four days. The collected nubbins were washed twice with 0.2 μm filtered seawater. Coral mucus was then “milked” from each nubbin^16^ into a 1.5 mL microfuge tube for 5 min, after discarding the initial 30 s worth of mucus release. Coral tissue was collected by spraying the same coral samples with an airbrush, containing 5 mL 10x TE buffer (10 mM Tris-HCl, pH 8.5, 1 mM EDTA, pH 8.0). The coral tissue slurry was collected in a sterile bag and transferred to a 1.5 mL microfuge tube. A total of 96 replicate coral tissue (n = 48) and mucus (n = 48) samples, and 16 filtered seawater samples were collected for DNA extraction. All extracted coral samples were stored at −20 °C until DNA extraction.

### *In situ* experiment

An *in situ* field manipulation experiment was conducted in Nan-wan, Kenting (21°57′108″N, 120°46′222″E) to complement the laboratory results. Three colonies of *A. muricata*, each at depth 8–10 m, were chosen for experimentation. The experiment began on December 03 2013 (winter season) and June 18 2014 (summer season), with each experimental run lasting 10 days. Experimental runs were carried out in the winter and summer to compare the difference in seawater temperature between the two seasons, and the interactive impact of water flow and temperature on bacterial community structure in the coral host. Each coral colony was partially enclosed in a clear plastic mesh box, with clearance in the mesh measuring 0.25 cm^2^ to reduce the water flow past the enclosed portion of the colonies (). Clear mesh netting was used to minimize the shading effect across the coral colony; this netting was checked daily to ensure that no algae were growing on it.

One coral sample (~2 cm) was collected from each coral colony on the first day of deployment. After that, two samples (~2 cm) were collected from each colony (one from enclosed, one from un-enclosed) on each of Day 5 and 10. One liter of seawater was also collected from the surrounding reef water on days when coral samples were gathered. A total of 30 coral samples and six water samples were collected for DNA analysis. Extraction of coral tissue (n = 30) and mucus (n = 30) followed the exact procedure as in the laboratory experiment. All extracted coral samples were stored at −20 °C until DNA extraction.

### Temperature, water flow and coral health physiology monitoring

A Sontek^®^ microADV (Acoustic Doppler Velocimeter) was used to measure the water flow in both the laboratory and *in situ* experiments. In the *in situ* experiment, the microADV measured the difference in the water velocity around the enclosed and non-enclosed portions of the coral colonies. Water temperature was monitored daily with a portable temperature meter (Hanna Instruments HI991003), and HOBO^®^ loggers. In the laboratory experiment, a HOBO^®^ logger was deployed in each tank, while six loggers were deployed around the coral colonies, one each on the inside and outside the enclosure, to monitor the temperature and irradiance.

A Walz^®^ diving-pulse amplitude modulated (PAM) fluorometer was used to monitor the change in coral photosystem health, using a 0.8 s saturating pulse of >4500 μmol photons m^−2^ s^−1^ and gain value of 12. In the laboratory experiment, three dark-adapted yield values (*F*_*v*_*/F*_*m*_) were obtained from three randomly chosen coral nubbins from each tank, an hour after the light was turned off. In the *in situ* experiment, three dark-adapted yield measurements (*F*_*v*_*/F*_*m*_) *pe*r branch were obtained from three randomly chosen branches of the enclosed and non-enclosed portions of each coral colony, half an hour after sunset, on sample collection days.

### DNA extraction, 16S rRNA gene sequencing and analysis

Genomic DNA was extracted using the phenol/chloroform/isoamyl alcohol method^87^. The pellet from the coral mucus was homogenized with 600 μL 10x TE buffer, and incubated with 30 μL 10% SDS and 10 μL 100 μg/mL RNase A for 30 min at 37 °C. A further 3 μL of proteinase K (20 mg/mL) were added and incubated at 50 °C for 45 min. The final incubation was to add 100 μL 5 M NaCl and 80 μL CTAB/NaCl solution, and incubate for another 10 min at 65 °C. Final DNA was extracted using chloroform/isoamyl alcohol (24:1), and phenol/chloroform/isoamyl alcohol (25:24:1). Final DNA was precipitated in 0.6x cold 2-propanol and centrifuged for 8 min at −20 °C. The genomic DNA solution was then transferred to a clean tube and stored at −20 °C before gene amplification. Seawater DNA extraction followed the same procedure as that for coral tissue and mucus.

Extracted DNA was amplified using 16S primers 968F (5′-AAC GCG AAG AAC CTT AC-3′) and 1391R (5′-ACG GGC GGT GWG TRC-3′). The expected DNA band (~423 bp) was cut from the agarose gel and the DNA was recovered by electroelution^87^. A total of 112 and 66 unique tags were used in the laboratory and *in situ* field experiments, respectively, to tag each of the PCR products of the bacterial V6-V8 region from different samples^81^. Amplicons were sequenced using Illumina Miseq (Yourgene Bio science, Taipei). A total of 3,687,775 and 3,456,572 sequences were generated for the laboratory and *in situ* field experiments, respectively, and processed through the MOTHUR software package. Sequences of length <280 bp, homopolymer runs exceeding 8 bp, and qscore <27 were removed. Chimerical reads were also removed using UCHIME^88^. OTUs were then classified according to their taxonomic affiliations of the V6-V8 sequences (laboratory experiment – 536,001 sequences; *in situ* field experiment – 243,589 sequences) using Ribosomal Database Project (RDP) Classifier software (v2.10, http://sourceforge.net/projects/rdp-classifier/) with a bootstrap value of 0.8, and compiled at each taxonomic level into a counts file for statistical analysis. Sequences were submitted to the NCBI Sequence Read Archive under accession numbers SRP062848 and SRP062884.

Multivariate statistical software with PERMANOVA (PRIMER v6) was used to measure the degree of similarity between the bacterial communities at the order level in the experiments^89^. A pairwise t-test was used to further determine the significant differences between the temperature treatments within the bacterial order groups. Counts for the bacterial communities were converted to relative abundance for analysis, and Bray-Curtis similarity was calculated between samples after the data were square-root-transformed. Normalized relative abundance of *Vibrio* spp. was further analyzed (PERMANOVA; PRIMER v6) to explore the changes in relative abundance of the potential pathogen when the coral host is under thermal stress. The normalized data for photochemical efficiency (F_v_/F_m_) of the symbiotic dinoflagellates were analyzed using one-way analysis of variance (ANOVA) to determine if significant differences were present between treatments in both the laboratory and *in situ* experiments.

## Additional Information

**How to cite this article**: Lee, S. T. M. *et al*. Water flow buffers shifts in bacterial community structure in heat-stressed *Acropora muricata. Sci. Rep.* **7**, 43600; doi: 10.1038/srep43600 (2017).

**Publisher's note:** Springer Nature remains neutral with regard to jurisdictional claims in published maps and institutional affiliations.

## Supplementary Material

Supplementary Figures
