# Supplementary material for: Water flow buffers shifts in bacterial community structure in heat-stressed Acropora muricata
Source: Sci Rep. 2017 Feb 27;7:43600. doi: 10.1038/srep43600 (PMC5327421; doi:10.1038/srep43600)
Supplement: Supplementary Figures [file srep43600-s1.pdf]

Water flow buffers shifts in bacterial community structure in heat-stressed *Acropora muricata*

Sonny T.M. Lee[1, 4, *], Simon K. Davy[2], Sen-Lin Tang[3, *], Paul S. Kench[1]

[1] School of Environment, The University of Auckland, Private Bag 92019, Auckland New Zealand.

[2] School of Biological Sciences, Victoria University of Wellington, Kelburn Parade, Wellington, New Zealand.

[3] Microbial Lab, Biodiversity Research Center, Academia Sinica, Taipei, 115, Taiwan.

[4] Department of Medicine, The University of Chicago, Chicago, IL, United States

[*] corresponding.author: STML: leet1@uchicago.edu, SLT: sltang@gate.sinica.edu.tw

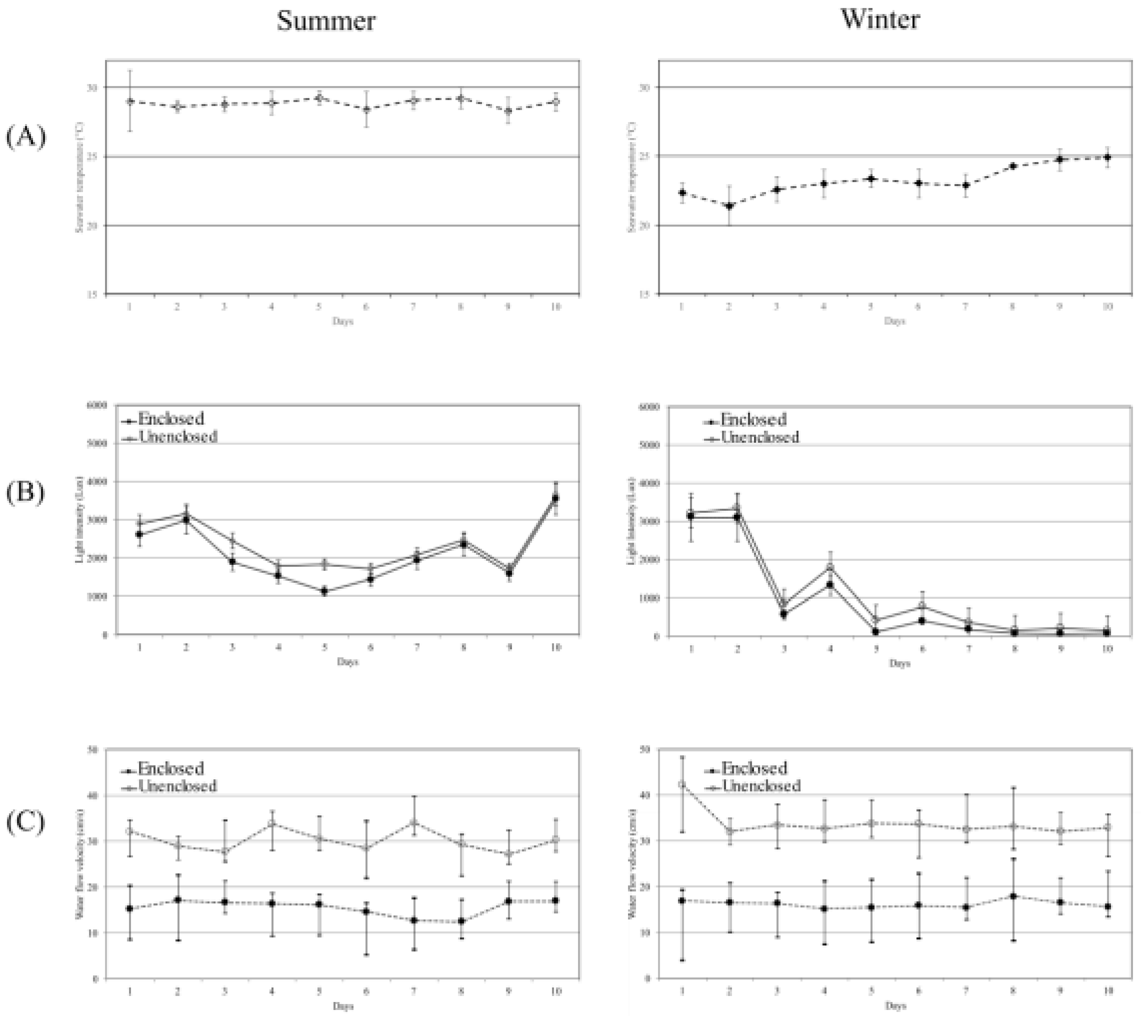

Figure S1. (A) Seawater temperature (Mean ± S.D.) during the summer and winter at the field site (Nanwan, Kenting, Taiwan) where the treatment coral colonies were located. (B) Light intensity and (C) water flow velocity of the three treatment colonies (Mean ± S.D.) during summer and winter.

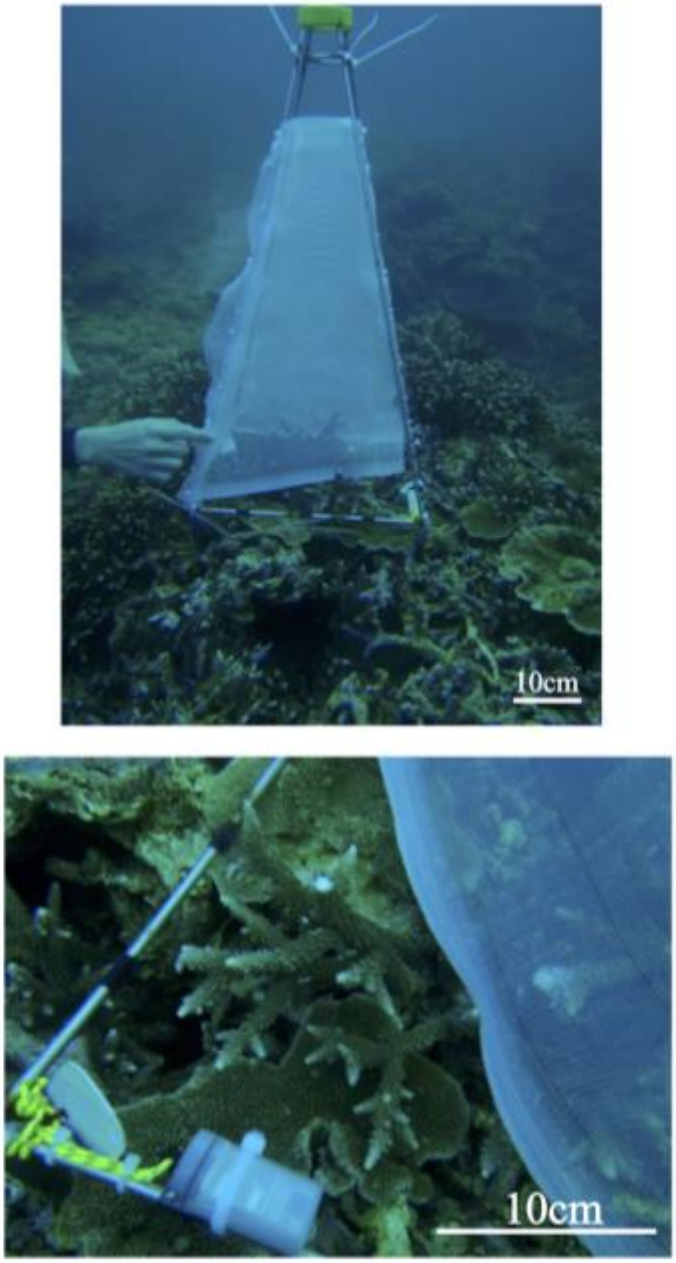

Figure S2. Three *Acroproa muricata* colonies were partially enclosed in a clear plastic mesh box, with clearance in the mesh measuring 0.25 cm^2 to reduce water flow past the enclosed portion of the colonies.
